# Intestinal Lymph Flow, and Lipid and Drug Transport Scale Allometrically From Pre-clinical Species to Humans

**DOI:** 10.3389/fphys.2020.00458

**Published:** 2020-05-21

**Authors:** Natalie L. Trevaskis, Given Lee, Alistair Escott, Kian Liun Phang, Jiwon Hong, Enyuan Cao, Kasiram Katneni, Susan A. Charman, Sifei Han, William N. Charman, Anthony R. J. Phillips, John A. Windsor, Christopher J. H. Porter

**Affiliations:** ^1^Drug Delivery, Disposition and Dynamics, Monash Institute of Pharmaceutical Sciences, Parkville, VIC, Australia; ^2^ARC Centre of Excellence in Convergent Bio-Nano Science and Technology, Monash Institute of Pharmaceutical Sciences, Parkville, VIC, Australia; ^3^Centre for Drug Candidate Optimisation, Monash Institute of Pharmaceutical Sciences, Parkville, VIC, Australia; ^4^Surgical and Translational Research Centre, Faculty of Medical and Health Sciences, The University of Auckland, Auckland, New Zealand; ^5^HBP/Upper GI Unit, Department of General Surgery, Auckland City Hospital, Auckland, New Zealand; ^6^Applied Surgery and Metabolism Laboratory, School of Biological Sciences, The University of Auckland, Auckland, New Zealand

**Keywords:** lymph, lymphatic, flow, lipid, drug, intestine, allometric, human

## Abstract

The intestinal lymphatic system transports fluid, immune cells, dietary lipids, and highly lipophilic drugs from the intestine to the systemic circulation. These transport functions are important to health and when dysregulated contribute to pathology. This has generated significant interest in approaches to deliver drugs to the lymphatics. Most of the current understanding of intestinal lymph flow, and lymphatic lipid and drug transport rates, comes from *in vitro* studies and *in vivo* animal studies. In contrast, intestinal lymphatic transport studies in human subjects have been limited. Recently, three surgical patients had cannulation of the thoracic lymph duct for collection of lymph before and during a stepwise increase in enteral feed rate. We compared these data to studies where we previously enterally administered controlled quantities of lipid and the lipophilic drug halofantrine to mice, rats and dogs and collected lymph and blood (plasma). The collected lymph was analyzed to compare lymph flow rate, triglyceride (TG) and drug transport rates, and plasma was analyzed for drug concentrations, as a function of enteral lipid dose across species. Lymph flow rate, TG and drug transport increased with lipid administration in all species tested, and scaled allometrically according to the equation A = *a*M^*E*^ where A is the lymph transport parameter, M is animal body mass, *a* is constant and *E* is the allometric exponent. For lymph flow rate and TG transport, the allometric exponents were 0.84–0.94 and 0.80–0.96, respectively. Accordingly, weight normalized lymph flow and TG mass transport were generally lower in larger compared to smaller species. In comparison, mass transport of drug via lymph increased in a greater than proportional manner with species body mass with an exponent of ∼1.3. The supra-proportional increase in lymphatic drug transport with species body mass appeared to be due to increased partitioning of drug into lymph rather than blood following absorption. Overall, this study proposes that intestinal lymphatic flow, and lymphatic lipid and drug transport in humans is most similar to species with higher body mass such as dogs and underestimated by studies in rodents. Notably, lymph flow and lipid transport in humans can be predicted from animal data via allometric scaling suggesting the potential for similar relationships with drug transport.

## Introduction

Following ingestion, dietary lipids, lipid soluble vitamins, and highly lipophilic drugs are assembled into triglyceride-rich lipoproteins in the enterocytes and transported from the intestine to the systemic circulation via the lymphatic system (Dixon, 2010; Trevaskis et al., 2015; Bernier-Latmani and Petrova, 2017; Cifarelli and Eichmann, 2019). In addition, the lymphatic system is a drainage system that collects interstitial fluid and returns it to the blood circulation, and a trafficking route for immune cells that surveil for disease-causing entities (i.e., foreign antigens) (Dixon, 2010; Trevaskis et al., 2015; Bernier-Latmani and Petrova, 2017; Cifarelli and Eichmann, 2019). The functions of the lymphatic system are important to health and when dysregulated can contribute to a range of pathologies including obesity, cardiovascular diseases, inflammatory diseases, acute and critical illnesses, and cancers (Alitalo, 2011; Trevaskis et al., 2015; Petrova and Koh, 2018). This has sparked significant interest in the lymphatic system as a therapeutic target, as well as approaches to deliver drugs to the lymphatics (Trevaskis et al., 2015; Maisel et al., 2017).

Most of our current understanding of intestinal lymph flow and the transport of lipids, drugs, and immune cells comes from *in vitro* studies and *in vivo* animal studies. Crucially, there is limited understanding of how well-animal studies reflect lymphatic transport in human subjects as the collection and study of human lymph is difficult (Trevaskis et al., 2015; Wang et al., 2016). Cannulation and external drainage of thoracic duct lymph has been performed in human patients for indications such as reduction of organ rejection after transplantation and treatment of inflammatory diseases (Wang et al., 2016). Knowledge of thoracic lymph flow and composition in human patients has largely come from these studies (Blomstrand and Dahlback, 1960; Schlierf et al., 1969; Wang et al., 2016). However, most of these were conducted more than 40 years ago (Wang et al., 2016). The study of lymphatic drug transport in humans is even more limited which makes it very difficult to translate preclinical data to the human situation. One small study attempted to quantify lymphatic transport of testosterone undecanoate in one patient after oral administration in 2.4 ml of arachis oil (Horst et al., 1976). The lymphatic recovery of testosterone undecanoate in this study was 8.7% of the dose over 10 h (Horst et al., 1976). This compares to recovery of 2.8–3.3 and 1.9% of the dose of testosterone undecanoate in lymph after administration to greyhound dogs and rats in similar commercial formulations (Shackleford et al., 2003; Hu et al., 2016).

Interestingly, the lymphatic transport of testosterone undecanoate was highest in human subjects and lowest in rats, with dogs between these. Similarly, we reported that lymphatic uptake of the lipophilic drug halofantrine is in the order dogs > rats > mice (Trevaskis et al., 2013). The lymphatic transport of CP532,623, CP524,515, and methylnortestonerone undecanoate was also higher in dogs than rats (White et al., 2009; Trevaskis et al., 2010a, b). This led us to speculate that there is a relationship between species size and lymphatic transport of fluid, lipids, and drugs. Such a relationship is important to confirm as it would facilitate prediction of lymphatic transport in humans from animal data.

Recently, three surgical patients had cannulation of the thoracic lymph duct for collection of lymph before and during a stepwise increase in enteral lipid feed rate. Here we (i) characterize thoracic lymph flow and lipid composition in these human subjects as a function of enteral lipid feed rate, (ii) compare lymph flow, lipid and drug transport between species of different mass, and (iii) develop scaling models to predict intestinal lymph flow, and lymph lipid and drug transport in human subjects. We find that lymph flow, and lymph lipid and drug transport scale allometrically according to the equation A = *a*M*^*E*^* where A is the lymph transport parameter (i.e., lymph flow rate or mass transport of TG or drug in lymph), M is animal body mass, *a* is constant and *E* is the allometric scaling exponent. We propose a mechanistic explanation for this and suggest that data from animal studies could be used to predict lymphatic transport parameters in humans via allometric scaling.

## Materials and Methods

### Materials

Halofantrine base (Hf) (1,3-dichloro-alpha-[2-(dibutylamino) ethyl]-6-(trifluoromethyl)-9-phenanthrenemethanol), 2,4-dichloro-6-trifluoromethyl-9-{1-[2-(dibutylamino) ethyl] phenanthrenemethanol HCl, and *N, N*-dibutyl-3-(1,3-dichloro-6-trigluoromethyl9-phenanthyl-3-hydroxypropionamide) (the internal standards for HPLC and HPLC–MS assays, respectively) were kind gifts from GlaxoSmithKline, King of Prussia, PA, United States. Oleic acid (OA), sodium chloride, ammonium sulfate, soybean oil, cremophor EL, triacetin (Sigma Chemicals, Australia), Tween 80 (BDH Chemicals, Australia), Maisine 35-1 (Gattefossé, France), Miglyol 812 (Sasol, Germany), Intralipid^®^ and normal saline for injection (Baxter Healthcare, Australia) were used as received. Acetonitrile, sodium dodecyl sulfate, glacial acetic acid, and ammonium hydroxide were analytical grade. Water was obtained from a Milli-Q (Millipore, Milford, MA, United States) purification system. Ketamine and Xylazine (Ilium Ketamil^®^, and Xylazil^®^, Troy laboratories) and Acepromazine (ACP-10 injection, Ceva Animal Health, Australia) were used for anesthesia. Triglyceride kit^®^ (Roche diagnostics GmbH, Mannheim, Germany) or Serum Triglyceride Determination kit (TR0100, Sigma Aldrich, United States) were used for analysis of TG levels. Polyethylene (PE) and polyvinyl chloride (PVC) cannulas with 0.96 and 0.58 mm, 0.8 and 0.5 mm, and 0.5 and 0.2 mm external and internal diameters were obtained from Microtube Extrusions, North Rocks, NSW, Australia. All other chemicals were analytical reagent grade.

### Experiment Design

Thoracic lymph fluid was collected from human patients before and during a step-wise increase in enteral feeding rate (containing lipid) and analyzed to determine thoracic duct lymph flow and lipid (TG) transport. These results were compared to data from previous studies where we enterally administered controlled quantities of lipid and the lipophilic drug halofantrine to mice, rats and dogs and collected either mesenteric lymph (for mice and rats) or thoracic lymph (for dogs) and blood/plasma (Trevaskis et al., 2013). The collected lymph was analyzed to compare lymph flow rate, and TG and drug (halofantine) transport rates as a function of enteral lipid dose across species. Importantly, we have recently demonstrated that the majority (60–90%) of the fluid and TG in thoracic lymph is sourced from the mesenteric lymph (Yadav et al., 2018) such that intestinal lymph flow, lipid and drug transport can be assessed via collection and comparison of mesenteric or thoracic lymph. Whilst data from the studies in mice, rats and dogs has been reported previously, albeit in a slightly different format, (Trevaskis et al., 2013) here we report the allometric scaling of the transport data for the first time.

### Human Lymph Collection Experiments

Human lymph collection studies were approved by the New Zealand Health and Disability Ethics Committees (Approval 12/NTB/67). Thoracic lymph fluid was collected from three male patients (weight 92, 98, 88 kg) via a 5 Fr cholangiography catheter (Endoscopic Cholangiography Set, ESC 500 Tuohy, Cook Medical, Bloomington, IN, United States) inserted into the thoracic lymph duct during an Ivor Lewis oesophagogastrectomy to resect an adenocarcinoma of the distal esophagus. The patients were well aside from the adenocarcinoma. The patients were fasted the day of surgery but received 50 ml of cream intraoperatively to facilitate identification and cannulation of the lymph duct. Lymph fluid was collected into tared tubes every 12 h for 1–2 h until days 3, 4, or 5 following surgery as shown in Table 1. The patients were fasted before surgery and for 24 h after surgery. They then received Nutrison standard (1.0 kcal/mL, Nutricia, New Zealand) feed via the proximal jejunum at different rates as outlined in Table 1. Nutrison standard enteral feed contains 3.9 g/dL total fat with 1 g/dL saturated fat, 2.2 g/dL monounsaturated fat and 0.7 g/dL polyunsaturated fat. For patient 1 feeding was stopped on day 3 (due to embolization), patient 2 on day 4 and patient 3 on day 5. Lymph flow rate was determined gravitrimetrically. TG concentration in the lymph samples was measured using the Serum Triglyceride Determination kit (TR0100, Sigma Aldrich, United States).

**TABLE 1:** Enteral feed rates in ml/h from days 1 to 5 in the three patients.

		Patient 1	Patient 2	Patient 3
	Weight	92 kg	98 kg	88 kg
Day 0	Surgery	0	0	0
Day 1	D1A1	0	0	0
	D1A2	0	0	0
	D1P	0	0	0
Day 2	D2A1	0	0	0
	D2A2	20	0	20
	D2P	20	0	20
Day 3	D3A1	Ceased	0	40
	D3A2		20	80
	D3P		20	80
Day 4	D4A1		20	80
	D4A2		40	80
	D4P		40	
Day 5	D5A1		40	
	D5A2		80	
	D5P		80	

Patients received Nutrison standard (1.0 kcal/mL and 3.9 g/dL total fat) feed via enteral tube into the proximal jejunum. *D# = day number (e.g., D1 = day 1), A1 = morning (am) sample 1 taken from 0700 to 0800, A2 = morning (am) sample 2 taken from 0800 to 0900, P = evening (pm) sample taken from 1900 to 2000.

### Animal Experiments

All surgical and experimental procedures were approved by the local institutional animal ethics committee and conducted in accordance with the Australian and New Zealand Guidelines for the Care and Use of Animals in Research. The methods and some results for the animal studies have been reported previously (Porter et al., 1996a, b; Caliph et al., 2000; Khoo et al., 2001, 2003; Trevaskis et al., 2013). Here we re-analyze the data to compare to the human subjects and develop an allometric model to describe intestinal lymph flow, and lymphatic transport of lipids and drugs. The studies were performed in male C57BL/6 mice (22–26 g), male Sprague-Dawley rats (280–320 g), and male greyhound dogs (∼32 kg). The rats and mice were anesthetised during mesenteric lymph collection whereas the dogs were conscious and freely moving during thoracic lymph collection. The mice were administered formulations into the duodenum over 1 h consisting of 1.6 mg/kg halofantrine and 18.1, 250, or 1000 mg/kg oleic acid dispersed in 0.5 ml of 0.2% Tween 80 in normal saline. The rats were infused with halofantrine lipid formulations at a rate of 2.8 ml/h for 2 h. The formulations consisted of ∼1.6 mg/kg halofantrine and 13.3, 18.1, 133, or 167 mg/kg oleic acid dispersed in 5.6 ml of 0.2% Tween 80 in normal saline, as described previously (Trevaskis et al., 2013). Dogs were fasted overnight and for 12 h post-dose, and administered a single (1 g) soft gelatin capsule containing 1.6 mg/kg halofantrine in 18.1 mg/kg long chain lipid in a SEDDs formulation (30.5% w/w soybean oil, 30.5% w/w Maisine 35-1, 31.6% w/w Cremophor EL, 7.4% w/w ethanol) (Khoo et al., 2003). Alternatively, the dogs were fasted or fed a can of commercial dog food containing ∼1000 mg/kg of fat 30–45 min prior to administration of 100 mg halofantrine base in standard tablet formulations (Khoo et al., 2001). After formulation administration the lymph fluid was collected continuously. Here we analyze data for 0–8 h following commencement of formulation administration. Lymph flow rates were determined gravitrimetrically. Lymph concentrations of TG were measured using commercial enzymatic kits and halofantrine concentrations were measured using HPLC or HPLC–MS as described previously (Porter et al., 1996a, b; Caliph et al., 2000; Khoo et al., 2001, 2003; Trevaskis et al., 2013).

### *In vitro* Metabolism of Halofantrine

Mouse, rat, dog and human intestinal microsomes and mouse, rat and human liver microsomes were obtained from Xenotech, Lenexa, KS. Dog liver microsomes were obtained from BD Gentest, Discovery Labware, Inc. Metabolic stability of halofantrine was assessed *in vitro* by incubating at 37°C with microsomes suspended in 0.1 M phosphate buffer (pH 7.4) at a final halofantrine concentration of 0.5 μM and microsomal protein concentration of 0.4 mg/mL (Doggett et al., 2012; Carosati et al., 2016).

Metabolic reactions were initiated by the addition of a NADPH-regenerating system (1 mg/mL NADP, 1 mg/mL glucose-6-phosphate, and 1 U/mL glucose-6-phosphate dehydrogenase) and MgCl_2_ (0.67 mg/mL) and were quenched by the addition of ice-cold acetonitrile at five time points (2, 5, 15, 30, and 60 min) (Doggett et al., 2012). Compounds were also incubated in the absence of NADPH to monitor for cofactor independent degradation in the microsomal matrix (2, 30, and 60 min). No compound loss was observed in the absence of NADPH. Quenched samples were centrifuged, and the clear supernatant was analyzed to monitor the extent of drug loss using the LC–MS assay described below. Concentration versus time data for each compound were fitted to an exponential decay function to determine the first order rate constant for substrate depletion, which was then used to calculate the degradation half-life and an *in vitro* intrinsic clearance (Cl_int_, *in vitro*) value (mL/min/mg microsomal protein).

A cocktail of known compounds (dextromethorphan, diclofenac, midazolam) was included in the incubation alongside test compounds, and the degradation half-life (min) values observed were in agreement with historical values, thereby validating the assay conditions employed (Obach, 1999; Doggett et al., 2012; Carosati et al., 2016).

Halofantrine concentrations in samples from the microsome metabolism studies were assayed using the same HPLC–MS assay as used for the mouse studies as described and validated previously (Trevaskis et al., 2013).

### Calculations and Modeling

#### Lymphatic Transport Data

The mass of TG and halofantrine transported in lymph was calculated from the product of the measured concentration and the lymph flow rate during each time period. These values were in some cases further normalized across species by dividing by body weight.

#### Allometric Analysis

To determine whether lymphatic transport parameters across species scaled allometrically (i.e., were related to body weight) according to the equation A = *a*M*^*E*^* (where A is the lymphatic transport parameter, M is animal body mass, *a* is a constant and *E* is the allometric scaling exponent), a plot of log A versus log M was prepared for each parameter using GraphPad Prism version 8.2.1 (GraphPad Software, Inc., La Jolla, CA, United States). A weighted (1/y^2^) non-linear regression line was fit through the data with gradient *E* and y intercept *a.* Goodness of fit was determined from the correlation co-efficient (r^2^) and weighted sum of squares, and the 95% confidence interval and standard error for the gradient *E* and y intercept *a* were determined.

Note that an *E* value of one indicates that the lymphatic transport parameter increases in direct proportion to the increase in body weight whereas *E* values less than one or greater than one indicate a less than or greater than proportional increase, respectively.

### Statistical Analysis

Statistics were analyzed using GraphPad Prism version 8.2.1 (GraphPad Software, Inc., La Jolla, CA, United States). The data are presented as mean ± standard error of the mean (SEM). Statistically significant differences among group means for different lipid doses or in different species were assessed by one-way analysis of variance (ANOVA) using a Tukey HSD *post hoc* test where variances were homogenous or Dunnett T3 *post hoc* test where variances were not homogenous. Differences with values of *p* < 0.05 were considered significant.

## Results

### Lymph Flow Rate and Triglyceride Transport Increase With Increases in the Quantity of Enteral Lipid Administered to Human Patients

**FIGURE 1:**
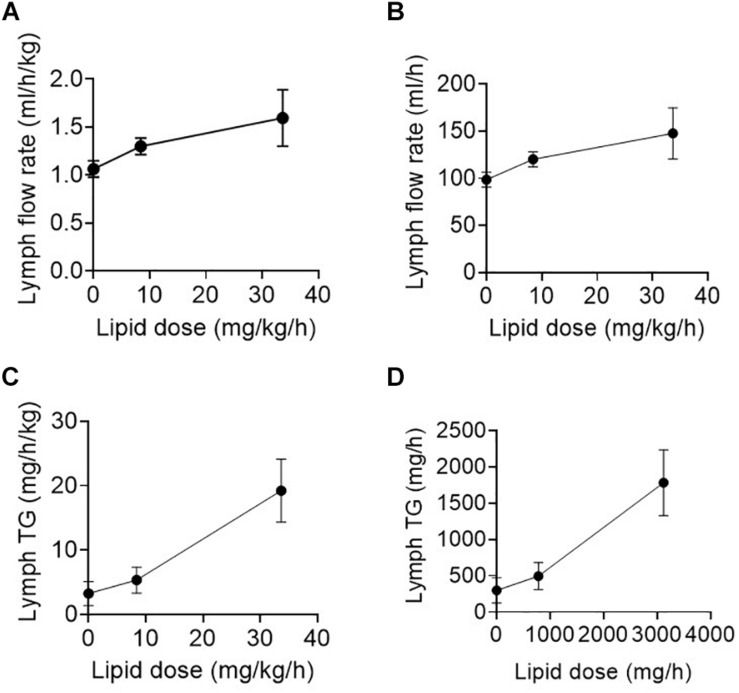
**(A,B)** Lymph flow rate (in ml/h/kg or ml/h) and **(C,D)** Lymph triglyceride (TG) transport rate (in mg/h/kg or mg/h) versus enteral lipid dose (in mg/h or mg/h/kg) in human patients infused with 0, 20, or 80 ml/h of enteral nutrition containing different lipid (TG) doses. Data shown are mean ± SEM for 19, 9, and 6 replicates from *n* = 3, 3, and 2 patients administered 0, 20, and 80 ml/h enteral nutrition, respectively.

The enteral feeding rate was increased in the surgical patients in step-wise fashion over several days with 0, 20, 40, and 80 ml/h of an enteral nutrition formula containing 0, 780, 1560, 3120 mg/h of TG (or 0, 8.4, 16.8, or 33.7 mg/h/kg of TG) (Table 1). Lymph flow rate and TG transport were measured at steady state for 1 h twice daily (i.e., at least 12 h after commencing the new infusion rate). Data for 0, 20, and 80 ml/h are shown as there were few replicates for 40 ml/h. The lymph flow rate increased significantly (*p* = 0.02) with increasing rate of lipid infusion from ∼99 ± 8 to 148 ± 27 ml/h or 1.1 ± 0.1 to 1.6 ± 0.3 ml/h/kg in the groups administered 0 and 80 ml/h of enteral nutrition, respectively (Figures 1A,B).

In addition to the increase in lymph flow rate, the rate of TG transport into lymph increased significantly (*p* < 0.01) with enteral nutrition from 3.2 ± 1.9 to 19.2 ± 4.9 mg/h/kg or 300 ± 174 mg/h to 1783 ± 454 mg/h in the groups administered 0 or 80 ml/h of enteral nutrition (containing 33.7 mg/h/kg or 3120 mg/h of TG) (Figures 1C,D). In the patients administered 80 ml/h enteral nutrition the rate of TG transport into lymph was thus approximately half of the rate at which TG was infused into the intestine.

### Lymph Flow Rate Across Species as a Function of Enteral Lipid Dose

**FIGURE 2:**
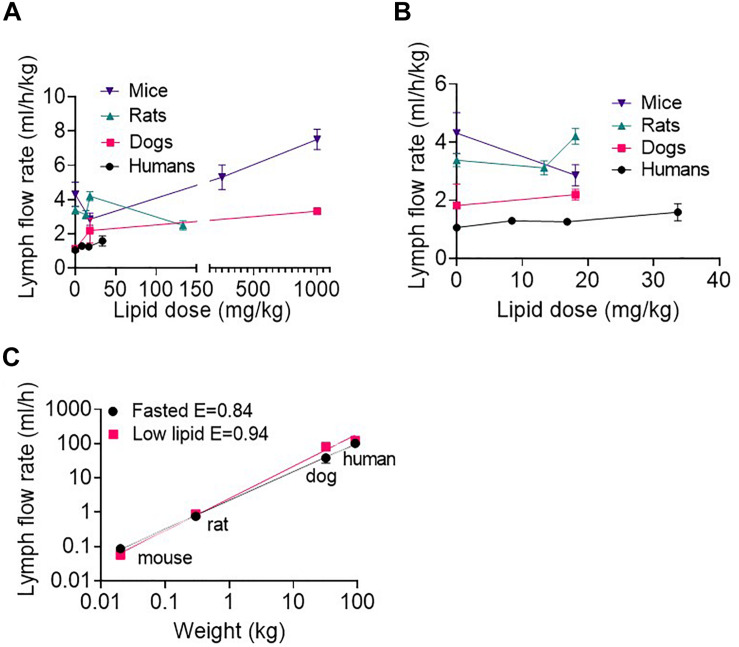
**(A)** Weight-normalized lymph flow rate (in ml/h/kg) at all lipid doses, and **(B)** Weight-normalized lymph flow rate (in ml/h/kg) at low lipid doses versus enteral lipid dose in mice, rats, dogs, and humans. **(C)** Allometric scaling of lymph flow rate (in ml/h) in the fasted state and after administration of low lipid doses of 8.4 mg/h/kg in humans and 18.1 mg/kg over 0–2 h in mice, rats and dogs. Mice and rats were intraduodenally infused with the lipid dose over 2 h, dogs were administered a single oral lipid dose and human patients were continuously infused with enteral nutrition at different rates. The lymph flow rates in mice, rats and dogs are the mean hourly rate over an 8 h lymph collection period whereas in human patients the lymph flow rates are the mean for 1 h collection periods completed twice daily. Data are mean ± SEM for *n* = 3–4 for mice, rats, and dogs and mean ± SEM for 19, 9, and 6 replicates from *n* = 3, 3, and 2 human patients administered 0, 20, and 80 ml/h enteral nutrition, respectively. Data for mice, rats and dogs are from published studies (Trevaskis et al., 2013) although this analysis has not previously been published.

Across species, the lymph flow rate generally increased with increasing enteral lipid administration (Figure 2A and Supplementary Table 1). The lymph flow rates in ml/h/kg (i.e., normalized to body mass) appeared slightly higher in mice than rats, rats than dogs and dogs than humans (Figures 2A,B and Supplementary Table 1), although there were some exceptions in rats where the lymph flow rate was relatively higher or lower than this (Figures 2A,B). Overall, the lymph flow rate increased less than proportionally with increases in species body mass. Accordingly, the lymph flow rate scaled allometrically (i.e., a plot of log lymph flow rate versus log body weight was linear) in the fasted state and after administration of low lipid doses (8.4 mg/h/kg in humans and 18.1 mg/kg over 0–2 h in animals) (Figure 2C). In the fasted state and after administration of the low lipid dose the exponents were 0.84 ± 0.03 and 0.94 ± 0.02, respectively, consistent with the less than proportional increase in lymph flow rate with species body weight (Figures 2A,B). An allometric plot could not be produced for other lipid doses as the administered lipid doses (mg/kg) differed across species.

### Lymph Triglyceride Transport Rate Across Species as a Function of Enteral Lipid Dose

**FIGURE 3:**
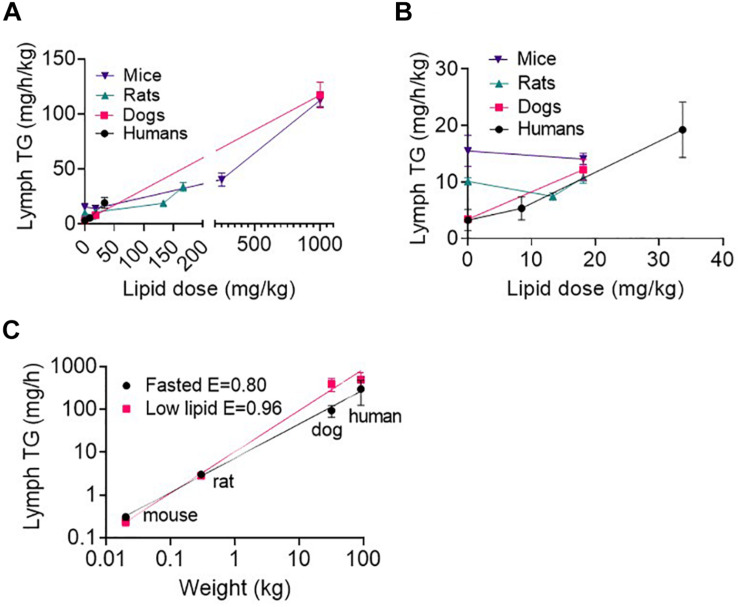
**(A)** Weight-normalized lymph triglyceride (TG) transport rate (in mg/h/kg) at all lipid doses, and **(B)** Weight-normalized lymph TG transport rate (in mg/h/kg) at low lipid doses versus enteral lipid dose in mice, rats, dogs, and humans. **(C)** Allometric scaling of lymph TG transport rate (mg/h) in the fasted state and after administration of a low lipid dose of 8.4 mg/h/kg in humans and 18.1 mg/kg over 0–2 h in mice, rats and dogs. Mice and rats were intraduodenally infused with the lipid dose over 2 h, dogs were administered a single oral lipid dose and human patients were continuously infused with enteral nutrition at different rates. The lymph TG transport rates in mice, rats, and dogs are the mean hourly rate over an 8 h lymph collection period whereas in human patients the lymph TG transport rates are the mean for 1 h collection periods completed twice daily. Data are mean ± SEM for *n* = 3–4 for mice, rats and dogs and mean ± SEM for 19, 9, and 6 replicates from *n* = 3, 3, and 2 human patients administered 0, 20, and 80 ml/h enteral nutrition, respectively. Data for mice, rats, and dogs are from published studies although this analysis has not previously been published (Trevaskis et al., 2013).

As expected, lymph TG transport rates generally increased with increasing enteral lipid infusion (Figure 3A and Supplementary Table 2). The exception was in mice and rats the lymph TG transport rate was slightly lower after administration of the small 13–18.1 mg/kg lipid doses compared to in the fasted state. Interestingly, in the fasted state (i.e., 0 ml/h/kg enteral lipid infusion) the lymph TG transport rate normalized for body mass was higher in mice than rats and in rats than dogs and humans (Figure 3B and Supplementary Table 2). In contrast, after administration of enteral lipid doses > 18 mg/kg the lymph TG transport rate normalized for body mass appeared similar across species (Figure 3A). Accordingly, the lymph TG transport rate (mg/h) scaled allometrically across species in the fasted state and after administration of the low lipid dose (8.4 mg/h/kg in humans and 18.1 mg/kg over 0–2 h in animals) (Figure 3C). In the fasted state and after administration of the low lipid dose the exponents were 0.80 ± 0.03 and 0.96 ± 0.05, respectively (Figure 3C). An allometric plot could not be produced for other lipid doses as the administered lipid dose differed across species. However, given the similarity in weight-normalized lipid transport for the mice and dogs at higher lipid doses (Figure 3A and Supplementary Table 2), the allometric exponent may be close to 1.0 at high lipid doses.

### Lymphatic Transport of Halofantrine Across Species as a Function of Enteral Lipid Dose

**FIGURE 4:**
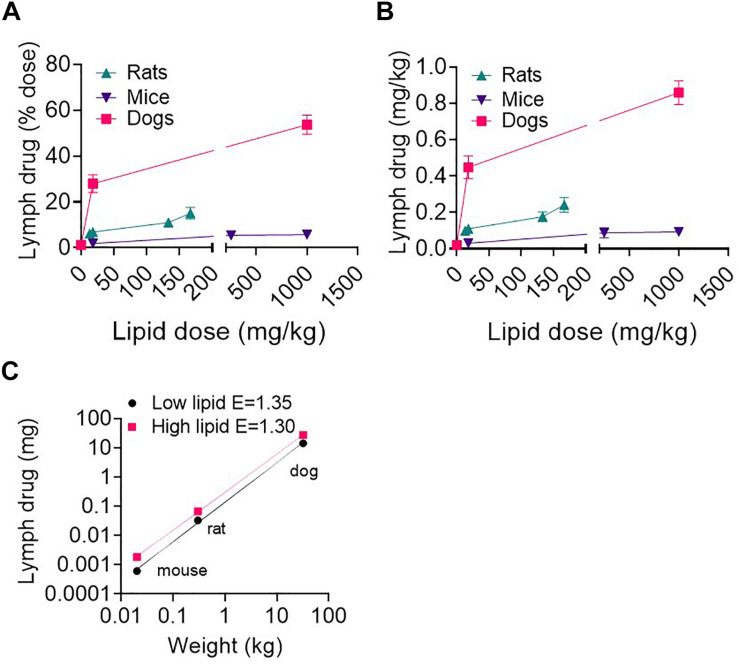
**(A,B)** Lymphatic transport of halofantrine (% dose or mg) versus enteral lipid dose in mice, rats, and dogs. **(C)** Allometric plots of lymphatic drug transport (mass transported) as a function of species body weight in mice, rats, and dogs administered 1.6 mg/kg drug (halofantrine) with a low lipid dose of 18.1 mg/kg or a high (>133 mg/kg) lipid dose. Data are mean ± SEM for *n* = 3–4. Data for mice, rats and dogs are from published studies (Trevaskis et al., 2013) although these results have not previously been presented as shown in **(C)**.

Mice, rats and dogs were enterally administered 1.6 mg/kg of the highly lipophilic drug halofantrine together with different lipid doses. As reported in a previous publication (Trevaskis et al., 2013), the lymphatic transport of halofantrine increased with lipid dose up to ∼130 mg/kg lipid and then appeared to plateau at a maximum in all species (Figures 4A,B and Supplementary Table 3). The lymphatic transport of halofantrine (mg/kg or as a% of dose administered) was higher in dogs than rats, and in rats than mice and therefore increased with species body mass (Figures 4A,B and Supplementary Table 3). This was despite the fact that lymph flow and TG transport increased less than proportionally with increasing species mass (i.e., E < 1). To our surprise there was an allometric relationship for the lymphatic transport of halofantrine (Figure 4C). Allometric plots for mass transport of halofantrine in lymph after administration of the low lipid dose (18.1 mg/kg) and for the maximum lymphatic transport of halofantrine after administration with high lipid doses (>160 mg/kg) had exponents of 1.35 ± 0.03 and 1.30 ± 0.02, respectively (Figure 4C).

### Lymphatic Transport of Highly Lipophilic Drugs and Prodrugs Increases Supra-Proportionally With Species Body Mass

**FIGURE 5:**
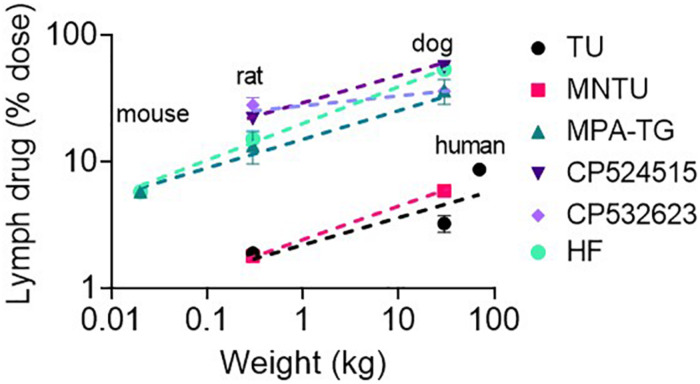
Lymphatic transport of lipophilic drugs and prodrugs following enteral administration to mice, rats, dogs, and/or humans as a function of species body weight (in kg). The model lipophilic prodrugs include testosterone undecanoate (TU), methylnortestosterone undecanoate (MNTU) and a di-palmitin prodrug of mycophenolic acid (MPA-TG). The model lipophilic drugs include CP524515, CP532623 and halofantrine (HF). Data for all compounds are for administration with a relatively high lipid dose (>130 mg/kg lipid). Data are taken from published studies (Horst et al., 1976; Shackleford et al., 2003; White et al., 2009; Trevaskis et al., 2010a, b; Han et al., 2014, 2016; Hu et al., 2016).

The lymphatic transport of halofantrine increased more than the increase in species body mass and scaled allometrically with an exponent > 1. Next we compared lymphatic transport data for a range of other lipophilic pro/drugs across species that we have measured previously after administration with a high lipid dose (>133 mg/kg) (Figure 5). All appeared to scale allometrically across species with an allometric exponent of 1.2–1.3 for dose-normalized mass transport of pro/drug in intestinal lymph (which results in an exponent of 0.2–0.3 for % pro/drug transport into lymph). This suggests that a similar allometric relationship would be seen for lymphatic transport of other highly lipophilic pro/drugs, and that lymphatic transport of pro/drugs in humans and dogs is likely to be significantly underestimated by studies in rodents.

### Why Does Lymphatic Drug and Prodrug Transport Increase With Species Body Mass?

Next we sought to determine why the lymphatic transport of pro/drugs increases supra-proportionally with species body mass and scales allometrically with an exponent of > 1 while lymph flow and mass transport of lipid in lymph increase sub-proportionally with species body mass and scale allometrically with exponents of < 1. We speculated that the greater than proportional increase in lymphatic pro/drug transport with species body mass could be due to increased drug absorption, decreased enterocyte-based drug metabolism, increased partitioning into intestinal lymph lipoproteins versus portal vein blood, or a combination thereof.

**FIGURE 6:**
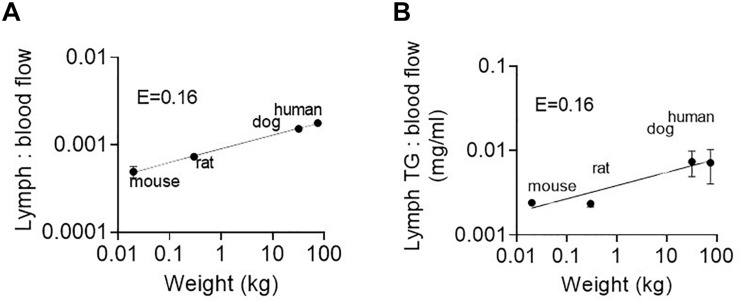
**(A)** Ratio of lymph flow rate to portal blood flow rate, and **(B)** Ratio of lymph TG transport rate to portal blood flow rate as a function of species body mass in mice, rats, dogs, and humans. Lymph flow and TG transport rate data are for administration of the low 8–18.1 mg/kg lipid dose in the current study. Portal blood flow rate data are taken from published data for the same weight (and strain) of mice (Xie et al., 2014), rats (Mansbach et al., 1991), dogs (Frink et al., 1992), and humans (Davies and Morris, 1993).

We suggest that the most likely explanation is increased partitioning into intestinal lymph versus portal vein blood. Partitioning into intestinal lymph occurs post-absorption and is driven by pro/drug partitioning into TG-rich lipoproteins in the enterocyte that are exclusively transported from the intestine via the lymphatic system (Trevaskis et al., 2015). The ratio of lymph flow rate or TG transport rate to portal blood flow rate is thus an important factor that drives differences in intestinal lymphatic drug transport. As shown in Figures 6A,B the ratio of lymph flow and lymph TG transport rate (from the current study) to portal blood flow rate [taken from published data for mice (Xie et al., 2014), rats (Mansbach et al., 1991), dogs (Frink et al., 1992), and humans (Davies and Morris, 1993)] is not constant across species (i.e., E ≠ 0) but rather increases with species body mass and scales allometrically with exponents of 0.16 ± 0.01 and 0.16 ± 0.04, respectively. This is because intestinal lymph flow and TG transport scale allometrically with exponents 0.9 and 0.96, respectively. In contrast, blood flow rate through the portal vein increases with an allometric exponent of 0.75–0.78 (Davies and Morris, 1993; Lindstedt and Schaeffer, 2002; Perelson and Wiegel, 2009). The net result is a greater increase in lymph flow compared to blood flow with an increase in body weight.

Differences in enterocyte-based metabolism was another potential explanation for the allometric scaling of lymphatic pro/drug transport across species. However, this was considered unlikely as we found that *in vitro* intrinsic clearance values of halofantrine are similar in intestinal microsomes across species (Supplementary Table 4). Additionally, we found that the lymphatic transport of a range of different pro/drugs with differing metabolic profiles scales similarly across species (Figure 5).

The increase in lymphatic pro/drug transport across species could also potentially be explained by differences in the efficiency of absorption of the lipophilic pro/drugs. We consider this unlikely as our data shows that lymphatic pro/drug transport scales with a very similar exponent for a range of pro/drugs across species and it is unlikely that absorption would scale with such similarity. We did attempt to profile the fraction of the dose absorbed across species via measurement of bioavailablity [as reported previously (Trevaskis et al., 2013)]. However, definitive comparison of absorption profiles is difficult since the contribution of pre-systemic metabolism to bioavailability is unknown.

## Discussion

Herein we report that lymph flow rate and TG transport increase with lipid administration and scale allometrically across species. Weight-normalized lymph flow and TG transport are lower in larger species (dogs, human) when compared to smaller species (rats, mice) (i.e., allometric exponents are < 1). In contrast, we show that intestinal lymphatic drug transport following enteral administration of several model highly lipophilic drugs and prodrugs increases supra-proportionally with species body weight (with an allometric exponent of 1.2–1.35). The increase in lymphatic drug transport across species is likely due to increased partitioning into intestinal lymph versus portal vein blood as the ratio of intestinal lymph flow and TG transport to blood flow increases with species body mass. The lymphatics may therefore be a more important route of absorption following oral administration of highly lipophilic drugs to larger species. Lymphatic drug transport is also most likely to be comparable in species with a body weight of similar magnitude such as humans and dogs. Conversely data obtained in rodents (as is most common) is likely to markedly underestimate lymphatic drug transport in larger animal species and humans. However, the fact that there is an allometric relationship for lymph flow, lipid and drug transport suggests that these parameters may be estimated in humans from animal studies.

In all species lymph flow rate generally increased with enteral lipid administration. An increase in lymph flow rate with enteral lipid administration has been reported previously in both *in vivo* lymph cannulation and *in situ* imaging models in rodents (Miura et al., 1987; Kassis et al., 2012). Lymph flow is controlled by various active (intrinsic) and passive (extrinsic) factors (Perelson and Wiegel, 2009; Kassis et al., 2012). The main intrinsic factor is phasic contraction of lymphangions (i.e., the segments of lymphatic vessels between valves) whereas extrinsic factors include the formation of lymph fluid, contractions of skeletal muscles in proximity to lymphatic vessels, cardiac and arterial pulsations, respiratory and central venous pressure fluctuations, and gastrointestinal peristalsis in the intestine (Gashev, 2008). Kassis et al. (2012) found that following intestinal lipid administration extrinsic factors dominate as the main force driving intestinal lymph flow and proposed that the extrinsic factor responsible for the increase in lymph flow with lipid administration is lymph formation.

We also found that lymph TG transport generally increases with enteral lipid administration, as would be expected since in the small intestine most lipids are assembled into TG-rich lipoproteins that are transported via the intestinal lymphatics (Kassis et al., 2012; Trevaskis et al., 2015). In all species, the mass of lipid transported in lymph was 40–60% lower than the mass of lipid administered enterally. Several previous studies in rodents have suggested that approximately 50% of long chain lipids are transported from the intestine via the lymph with the remaining transported via the portal vein (Bloom et al., 1951; Kiyasu et al., 1952; Mansbach et al., 1991; Trevaskis et al., 2013). The same is likely to be true in the dogs and human patients in our study. Historical studies in human patients with a thoracic duct cannula in place reported that the thoracic lymph lipid composition reflects dietary lipid and 19–27% of fed lipid was recovered in thoracic lymph (Blomstrand and Dahlback, 1960; Schlierf et al., 1969). We suspect that this value may be lower than expected due to incomplete collection of the lymph.

Interestingly, we found that in the fasted state the weight-normalized transport of TG into lymph (in mg/h/kg) was in the order mice > rats > dogs ≈ humans (and thus the allometric exponent was 0.8). In contrast, after infusion of higher lipid doses > 18 mg/kg the weight-normalized transport of TG was more similar across species (and thus the allometric exponent is closer to one). In the fasted state the lipids in intestinal lymph are endogenous in origin. The endogenous lipids may be sourced from bile, the intestinal blood supply or enterocyte cytosolic lipid droplets comprised of lipids stored from previous meals (Shiau et al., 1985; Mansbach and Nevin, 1998; Trevaskis et al., 2006; Porter et al., 2007). Rats and mice have continuous secretion of bile at a relatively constant rate as they lack a gall bladder whereas in dogs and humans, bile release from the gallbladder is intermittent and increases substantially when lipid is present in the intestine (Shiau et al., 1985; Porter et al., 2007). The increased lipids in lymph in the fasted state in mice and rats may thus reflect the continuous bile supply of endogenous lipids. The higher intestinal lymph flow rate in fasted mice and rats when compared to dogs and humans may also have been due to the increased supply of fluids and lipids to the intestine from bile.

Across species, both lymph flow and lipid transport scaled via a weight-based allometric relationship. In the fasted state the exponents for lymph flow and TG transport were 0.84 and 0.8, respectively, whereas after administration of 18.1 mg/kg lipid the exponents for lymph flow and lipid transport were 0.94 and 0.96, respectively. Previously, Perelson reported that thoracic lymph flow rate scales allometrically with an exponent of 0.89 (they did not assess lymphatic lipid or drug transport). The exponent of 0.89 was derived from data across nine mammalian species with body mass ranging from 0.09 kg (hamster) to 70 kg (human) (Perelson et al., 2006; Perelson and Wiegel, 2009). In this study, the fed or fasted state of each species was not described and may not have been controlled. The exponent was proposed to stem from the intrinsic and extrinsic forces that drive lymph flow. The extrinsic forces include periodic movement of the heart and lungs, peristaltic movement of the intestine and locomotion of the body (Perelson et al., 2006; Perelson and Wiegel, 2009). The heart, lungs and intestine move continuously whereas locomotion occurs during some fraction of the time. In general lymph movement arising from spontaneous muscle movement is expected to scale according to metabolic rate and hence scale with an exponent of 0.75, whereas lymph transport due to muscle contraction during locomotion is expected to scale with an exponent of 0.83 according to data for movement relative to body mass, as described in detail by Perelson (Perelson and Wiegel, 2009). In addition, lymph flow will be driven by lymph volume which scales in direct proportion to mass (i.e., exponent 1). It is thus difficult to precisely predict the scaling of lymph flow across species under different conditions (e.g., with movement, feeding, hydration, etc.). Our finding that lymph flow scales with exponents 0.84 and 0.94 in the fasted state and after infusion of a small amount of lipid is, however, consistent with the above discussion of factors driving lymph flow. The slight increase in the exponent with lipid infusion may be related to a greater contribution of extrinsic factors driving lymph flow such as intestinal peristalsis and lymph formation stemming from the infused lipids and fluids, as was suggested by Kassis et al. (2012). Our allometric exponents for lymph lipid transport (0.8–0.96) were also similar to that for lymph flow (0.84–0.94).

Perhaps most surprisingly, we found that the lymphatic transport of the highly lipophilic drug halofantrine scaled allometrically across mice, rats, and dogs with exponents of 1.3–1.35 after administration with low or high lipid doses. The lymphatic transport of halofantrine (as a % of the administered dose) was thus substantially higher in dogs than rodents. Notably, similar trends were also seen upon analysis of published data for other highly lipophilic compounds (Figure 5). The lymphatic transport of all drugs and prodrugs scaled with an allometric exponent of 1.2–1.3 for mass transport of pro/drug in lymph. This provides support that a similar allometric relationship would be seen for lymphatic transport of most drugs.

Using the allometric relationship from mice, rats, and dogs we predict that the lymphatic transport of halofantrine would be very high in human patients (44% of the dose after administration with a capsule containing 18.1 mg/kg lipid and maximum transport of up to 76% of the dose after administration with a large lipid dose such as a meal). The lymphatic recovery of halofantrine in humans is thus expected to be most similar to preclinical data obtained in larger animals such as dogs and approximately 4 fold higher than rats. The dog may therefore be a more representative species than rodents for estimating lymphatic drug transport in humans, as hypothesized previously (Shackleford et al., 2003; Trevaskis et al., 2010a). Alternatively, lymphatic transport in humans could be predicted from studies conducted in a couple of animal species via allometric scaling.

Also surprising was that the lymphatic transport of halofantrine (and other pro/drugs) appeared to increase significantly more with species size than did lymph flow and lipid transport (*E* = 1.2–1.35 for drug and *E* = 0.80–0.96 for lipid and flow). Additional studies and analyses were conducted to determine whether the increase in lymphatic drug (halofantrine) transport was related to increases in drug absorption, decreases in enterocyte-based drug metabolism or increases in drug partitioning into intestinal lymph versus portal vein blood. The data supported that an increase in drug partitioning into lymph rather than blood is the more important contributor, reflecting the increase in the ratio of intestinal lymph flow and TG transport to portal vein blood flow rate with increases in species size (Figures 6A,B).

There are several other implications of the increase in the ratio of intestinal lymph to blood flow with species size. Firstly, intestinal lymph may play a more important role in pathophysiological conditions in humans (Alitalo, 2011; Trevaskis et al., 2015; Petrova and Koh, 2018), than is predicted from studies in rodents. Secondly, Charman and Stella (1986) previously suggested that a log D of 5 is required for a drug to be a candidate for significant intestinal lymphatic uptake. This was calculated from an estimated 500:1 ratio of portal blood to intestinal lymph flow rate and because 1% of lymph consists of lipoprotein lipids. Since a drug must partition into these lipoprotein lipids in order to gain access to lymph, a drug must have a lipid to aqueous partition ratio of 50,000:1 or log D > 4.7 in order to access lymph in significant quantities. In human patients the portal vein blood flow rate is ∼1150 ml/min or 69,000 ml/h (Davies and Morris, 1993) and the thoracic lymph flow rate was 100–150 ml/h in our study depending on the lipid infusion rate. The ratio of lymph to blood flow is therefore approximately 460–690 in humans. In comparison, in mice portal vein blood flow rate is ∼116 ml/h and lymph flow rate in our study was 50–150 μl/h such that the ratio of portal blood to lymph flow is 780–2340. A higher relative log D may therefore be required to drive significant lymphatic uptake of a drug in small species such as mice.

## Conclusion

We show here that lymph flow and TG transport increase with increases in enteral lipid administration in mice, rats, dogs, and humans. Intestinal lymph flow and lipid transport scale allometrically with an exponent of 0.8–0.96 and thus weight-normalized intestinal lymph flow and lipid transport decreases with increasing species size. In contrast, weight normalized lymphatic drug transport increases with species body weight and scales allometrically with an exponent of 1.2–1.35 for mass transport in lymph. Intestinal lymph flow, and lymph lipid, and drug transport in human subjects are thus expected to be most similar to mammals of similar body weight and lymphatic drug transport is expected to be significantly underestimated (approximately 4 fold) in rodent studies. However, these parameters may potentially be predicted from studies in several species by allometric scaling. The increase in lymphatic drug transport with species body weight appeared to be due to increases in drug partitioning into intestinal lymph (rather than blood) because the ratio of intestinal lymph to blood flow is higher in larger species. The higher ratio of lymph to blood flow and transport in humans compared to rodents further supports the suggestion that lymphatic transport may potentially play a more important role in not only drug transport but also intestinal lymph-related pathophysiological conditions in humans such as inflammatory and metabolic diseases, cancers and acute and critical illness, than is predicted from studies in rodents.

## Data Availability Statement

All datasets generated for this study are included in the article/Supplementary Material.

## Ethics Statement

The studies involving human participants were reviewed and approved by New Zealand Health and Disability Ethics Committee. The patients/participants provided their written informed consent to participate in this study. The animal study was reviewed and approved by Monash Institute of Pharmaceutical Sciences Animal Ethics Committee.

## Author Contributions

NT designed and performed experiments, analyzed data, interpreted results, and drafted the manuscript. AE, KP, JH, AP, and JW designed the human experiments, assisted the human lymph collection, assisted with interpretation of the human data, and reviewed and edited the manuscript. GL analyzed the human data, interpreted human data and reviewed and edited the manuscript. EC performed HPLC–MS analysis, assisted with microsome experiments and reviewed and edited the manuscript. KK performed the microsome experiments and reviewed and edited the manuscript. SC designed the microsome experiments, helped interpret the results of all experiments and reviewed the manuscript. SH and WC helped design and interpret animal experiments and reviewed and edited the manuscript. CP designed experiments, interpreted results and reviewed and edited the manuscript.

## Conflict of Interest

CP, NT, and SH are inventors of a lymph-directing glyceride prodrug technology. Data for the prodrug dipalmitin-mycophenolic acid (MPA-TG) is described in the manuscript. This technology has been patented and licensed via a commercial agreement with PureTech Health, Boston. PureTech Health have subsequently entered into a collaboration agreement with Boehringer Ingelheim to explore the technology in immune modulation. The remaining authors declare that the research was conducted in the absence of any commercial or financial relationships that could be construed as a potential conflict of interest.
