# Supplementary material for: Intestinal Lymph Flow, and Lipid and Drug Transport Scale Allometrically From Pre-clinical Species to Humans
Source: Front Physiol. 2020 May 21;11:458. doi: 10.3389/fphys.2020.00458 (PMC7326060; doi:10.3389/fphys.2020.00458)
Supplement: Supplementary file 1 [file Data_Sheet_1.pdf]

Supplementary Table 1: Weight-normalised lymph flow rate (in ml/h/kg) at different enteral lipid dose rates (in mg/kg in animals or mg/h/kg in humans). Mice and rats were intraduodenally infused with the lipid dose over 2 h, dogs were administered a single oral lipid dose and human patients were continuously infused into the jejunum at the different rates. Data shown are mean ± SEM for n=3-4 for dogs, rats and mice and mean ± SEM for 19, 9 and 6 replicates from n=3, 3 and 2 human patients administered 0, 20 and 80 ml/h enteral nutrition, respectively. Data for mice, rats and dogs are from published studies (Trevaskis et al., 2013) although this analysis has not previously been published.

**Lipid dose**	**Humans**	**Dogs**	**Rats**	**Mice**
0	1.06 ± 0.09	1.14 ± 0.17	3.38 ± 0.23	4.31 ± 0.71
8.4	1.30± 0.09			
13.3			3.12 ± 0.24	
18.1		2.61 ± 0.22	4.20 ± 0.26	2.86 ± 0.36
33.7	1.59 ± 0.29			
133.3			2.51 ± 0.27	
250				5.31 ± 0.72
1000		3.33 ± 0.16		7.51 ± 0.60

Supplementary Table 2: Weight-normalised lymph triglyceride (TG) transport rate (in mg/h/kg) after administration of different enteral lipid doses (in mg/kg in animals or mg/h/kg in humans). Mice and rats were intraduodenally infused with the lipid dose over 2 h, dogs were administered a single oral lipid dose and human patients were continuously infused into the jejunum at the different rates. The lymph TG transport rates in mice, rats and dogs are the mean hourly rate over an 8 h lymph collection period whereas in human patients the lymph TG transport rates are the mean for one hour collection periods completed twice daily. Data shown are mean ± SEM for n=3-4 for dogs, rats and mice and mean ± SEM for 19, 9 and 6 replicates from n=3, 3 and 2 human patients administered 0, 20 and 80 ml/h enteral nutrition, respectively. Data for mice, rats and dogs are from published studies (Trevaskis et al., 2013) although this analysis has not previously been published.

**Lipid dose**	**Humans**	**Dogs**	**Rats**	**Mice**
0	3.23 ± 1.89	3.41 ± 0.58	10.12 ± 0.64	15.49 ± 2.73
8.4	5.35 ± 2.04			
13.3			7.43 ± 0.66	
18.1		7.95 ± 2.68	10.77 ± 0.97	14.06 ± 0.98
33.7	19.24 ± 4.90			
133.3			18.81 ± 2.07	
166.67			33.67 ± 4.02	
250				40.37 ± 5.99
1000		117.15 ± 11.66		112.46 ± 5.76

Supplementary Table 3: Lymphatic transport of halofantrine (% dose over 8 h) after administration of different enteral lipid doses (in mg/kg) in mice, rats and dogs. Data are mean ± SEM for n=3-4 animals. Data are from published studies (Trevaskis et al., 2013).

Lipid dose	Dogs	Rats	Mice
0	1.23 ± 0.35		
13.3		6.17± 0.93	
18.1	27.98 ± 3.85	6.89 ± 0.34	1.91 ± 0.35
133.3		11.01 ± 1.65	
166.67		15.10 ± 2.52	
250			5.49 ± 1.74
1000	53.69 ± 4.12		5.79 ± 0.51

Supplementary Table 4: Intrinsic clearance of halofantrine (µl/min/mg protein) calculated following *in vitro* incubation with microsomes derived from the intestine or liver of mice, rats, dogs and humans. Data are for n=2-3 replicates with mean ± standard deviation as shown.

**Replicate #**	**In Vitro CLint (µL/min/mg protein)**							
	**Liver microsomes**				**Intestinal microsomes**			
	**Human**	**Dog**	**Rat**	**Mouse**	**Human**	**Dog**	**Rat**	**Mouse**
1	23.1	8.4	20.4	27.0	15.1	13.6	6.8	35.5
2	22.4	5.7	11.2	26.5	13.2	15.6	8.2	35.4
3	30.7	7.4	7.5	27.7	---	---	---	---
**Mean**	**25.4**	**7.2**	**13.1**	**27.1**	**14.2**	**14.6**	**7.5**	**35.5**
**Stdev**	**4.6**	**1.4**	**6.7**	**0.6**	**NA**	**NA**	**NA**	**NA**
